# Serum proteome profiling of naturally acquired *Babesia rossi* infection in dogs

**DOI:** 10.1038/s41598-023-37312-9

**Published:** 2023-06-23

**Authors:** Josipa Kuleš, Ivana Rubić, Vladimir Farkaš, Renata Barić Rafaj, Jelena Gotić, Martina Crnogaj, Richard Burchmore, David Eckersall, Vladimir Mrljak, Andrew L. Leisewitz

**Affiliations:** 1 Department of Chemistry and Biochemistry, Faculty of Veterinary Medicine, University of Zagreb, Zagreb, Croatia; 2 Laboratory of Proteomics, Faculty of Veterinary Medicine, Internal Diseases Clinic, University of Zagreb, Zagreb, Croatia; 3 Glasgow Polyomics, College of Veterinary, Medical and Life Sciences, University of Glasgow, Glasgow, UK; 4 College of Veterinary, Medical and Life Sciences, School of Veterinary Medicine, University of Glasgow, Glasgow, UK; 5 Department of Clinical Sciences, Auburn University College of Veterinary Medicine, Auburn, AL USA; 6 Department of Companion Animal Clinical Studies, Faculty of Veterinary Science, University of Pretoria, Onderstepoort, South Africa

**Keywords:** Biochemistry, Molecular biology, Systems biology, Biomarkers, Molecular medicine

## Abstract

Babesiosis is a disease of significant medically and veterinary importance with worldwide distribution. It is caused by intra-erythrocyte protozoal parasites, with *Babesia rossi* causing the most severe clinical signs of all the large *Babesia* parasites infecting dogs. The disease can be clinically classified into uncomplicated and complicated forms with a wide range of clinical presentations from a mild, subclinical illness to complicated forms and death. The aim of this study was to assess serum proteomic profiles from dogs with babesiosis and healthy dogs using a label-based proteomics approach. Altogether 32 dogs naturally infected with *B. rossi* (subdivided into 18 uncomplicated cases and 14 complicated cases of babesiosis) and 20 healthy dogs were included. There were 78 proteins with significantly different abundances between the three groups of dogs. Elucidation of proteins and pathways involved in canine babesiosis caused by *B. rossi* have revealed key differences associated with haemostasis, innate immune system, lipid metabolism and inflammation. Shotgun proteomic profiling allowed identification of potential serum biomarkers for differentiation of disease severity in canine babesiosis caused by *B. rossi*. These findings may be applicable to the study of host-parasite interactions and the development of novel therapeutic targets.

## Introduction

Babesiosis is a widespread haemoprotozoan disease that can infect various vertebrate hosts, including humans. In dogs, babesiosis is an emerging tick-borne disease caused by distinct *Babesia* species. The Babesia parasites that most commonly infect dogs are *B. rossi*, *B. canis* and *B. vogeli*^1–3^. The life cycle of *Babesia* includes asexual multiplication in vertebrate blood cells, sexual reproduction in the vector and the production of sporozoites in the salivary glands of the vector^4^. After the injection of sporozoites with saliva during the tick bite, sporozoites penetrate directly into the erythrocyte and all the parasitic stages develop in erythrocytes. The parasite produces merozoites, which invade a new erythrocyte after erythrocyte lysis.

*Babesia rossi*, endemic in tropical and sub-tropical sub-Saharan Africa, is known to cause the most severe disease of all the *Babesia* species infecting dogs. Numerous studies of *B. rossi* infections in dogs have led to clinical and pathological descriptions of the disease^5,6^. Laboratory findings and clinical signs are used to classify the disease as uncomplicated or complicated, the latter being marked by the development of systemic inflammatory response syndrome (SIRS) and multiple organ dysfunction syndrome (MODS)^5,7,8^.

At presentation, dogs show various clinical signs including fever, lethargy, weakness, pale mucous membranes, icterus, splenomegaly and, more rarely, shock or central nervous system signs^9,10^. Because the clinical signs are common to other diseases, infection has to be diagnosed using a stained peripheral blood smear and confirmed by PCR.

The trademark manifestation of infection with *B. rossi* is haemolytic anaemia. Resulting decrease in oxygen-carrying capacity of the blood can lead to tissue hypoxia and subsequent organ dysfunction, impacting the overall severity and prognosis of the infection. This results in a range of clinical signs and complications, including lethargy, weakness, pale mucous membranes, icterus, and in severe cases, shock and organ dysfunction, such as acute kidney injury (AKI), cerebral babesiosis, acute respiratory distress syndrome (ARDS), pancreatitis, rhabdomyolysis, a consumptive coagulopathy, excessive pro-inflammatory response and myocardial dysfunction. These complications are associated with high morbidity and mortality^6,11–16^.

Host-parasite interactions are critical for invasion and for establishment of intracellular niches for parasite replication, differentiation, and persistence, as well as directly linked to pathogenicity and virulence which characterize symptomatic infections with this protozoan haemoparasites^17–19^. Early detection of complicated babesiosis is of importance in clinical practice for developing a therapeutic protocol as well as establishing a prognosis. Therefore, protein profiling in babesiosis is useful for understanding disease pathogenesis and the host immune response. New high-throughput omics technologies offer cutting-edge approaches to the discovery of a number of new potential disease-related biomarkers in canine babesiosis. Using a gel-based proteomic approach we previously identified several differentially expressed serum proteins in dogs with babesiosis due to *B. canis* infection. Identified proteins were associated with an inflammation-mediated acute phase response, complement and coagulation cascades, apolipoprotein and the vitamin D metabolism pathway^20,21^. To the authors’ knowledge, there are no proteomic studies that have examined dogs infected with *B. rossi*. The aim of this study was to compare the serum proteomes in healthy dogs and dogs with uncomplicated and complicated babesiosis caused by *B. rossi* using a label-based high-throughput proteomics approach. Bioinformatics tools were employed to elucidate altered biological pathways in babesiosis, and several candidate markers were validated by western blot.

## Results

### Proteomic profiling between the control group, uncomplicated and complicated babesiosis

**Figure 1:**
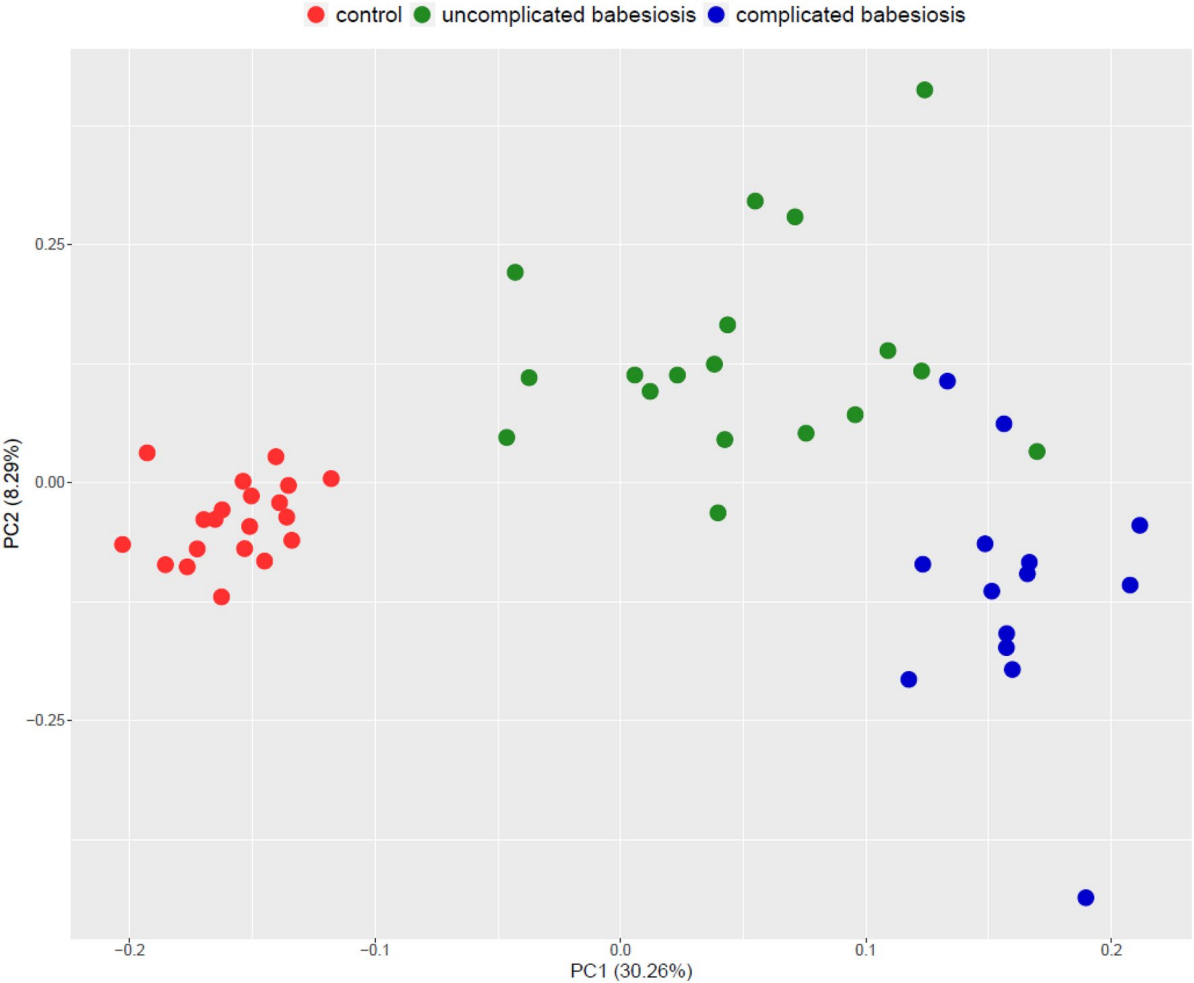
Principal component analysis (PCA) score plot showing the distribution of samples from control group (red dots), uncomplicated babesiosis (green dots) and complicated babesiosis (blue dots) group.

**Figure 2:**
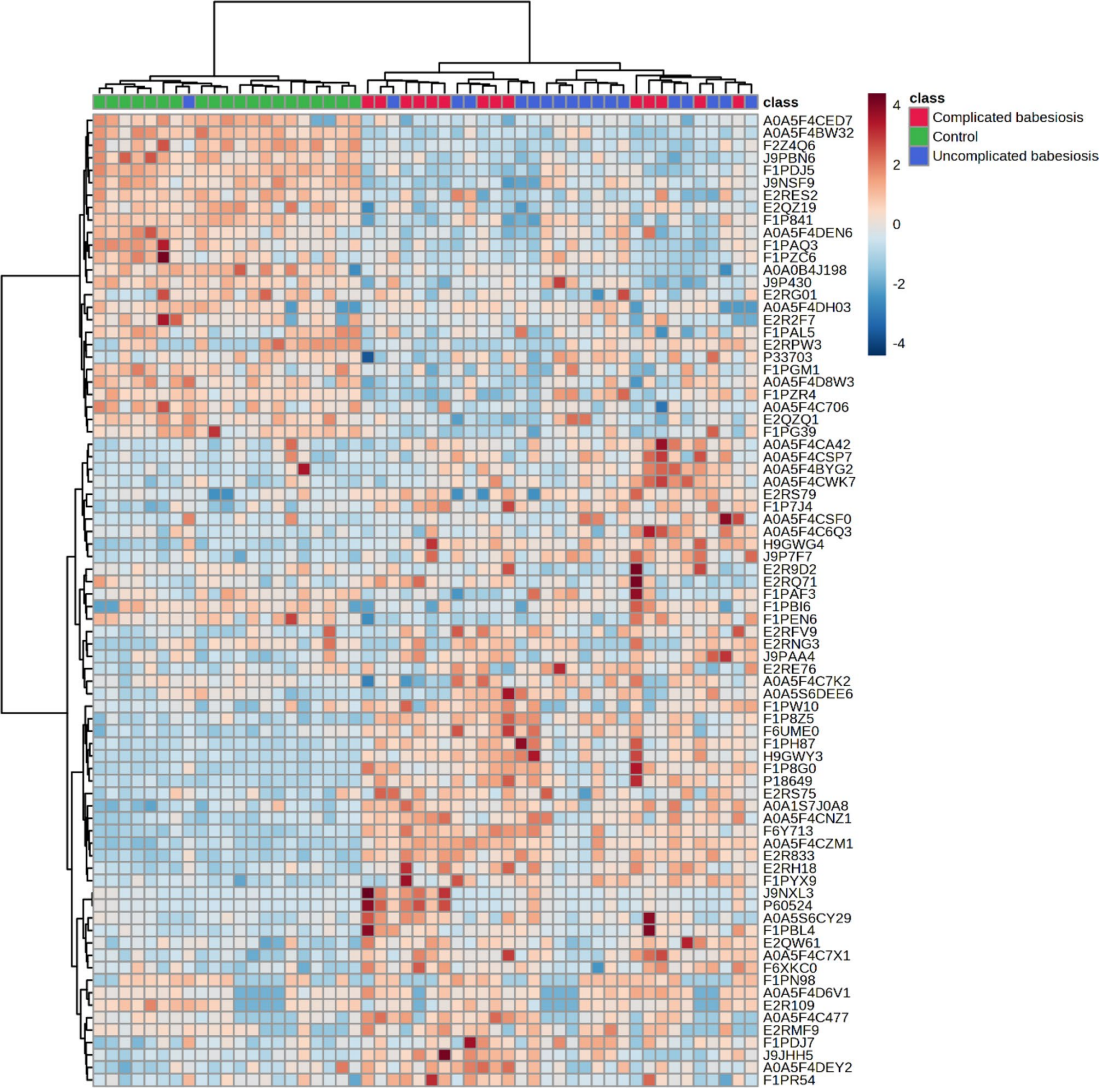
Hierarchical cluster analysis (HCA) based on the proteins with significantly differential abundances between control group (green panel), uncomplicated babesiosis (blue panel) and complicated babesiosis (red panel) using Euclidean as a distance measure and ward as a clustering algorithm. Each colored cell on the map corresponds to the abundance, with the red color meaning an increased, and blue a decreased abundance. Full protein names for accession IDs can be found in the Supplementary Table 1.

In this study, 128 proteins were identified and quantified by a label-based quantitative proteomic approach according to set criteria (at least 2 unique peptides and 1% FDR). In total, after excluding proteins having more than 50% missing values, there were 78 proteins with significantly different abundances between the control group, uncomplicated and complicated babesiosis, as accessed by the Kruskal–Wallis test (Supplementary Table 1). A principal component analysis (PCA) plot showed very clear discrimination of the groups (Fig. 1). The clusters were separated based on the principal component 1 (PC1), which captured 30.26% of the variance in the dataset, while PC2 captured 8.29% of the variance. Hierarchical cluster analysis (HCA) based on the proteins with significantly differential abundances between groups is presented as a heat map (Fig. 2).

**Figure 3:**
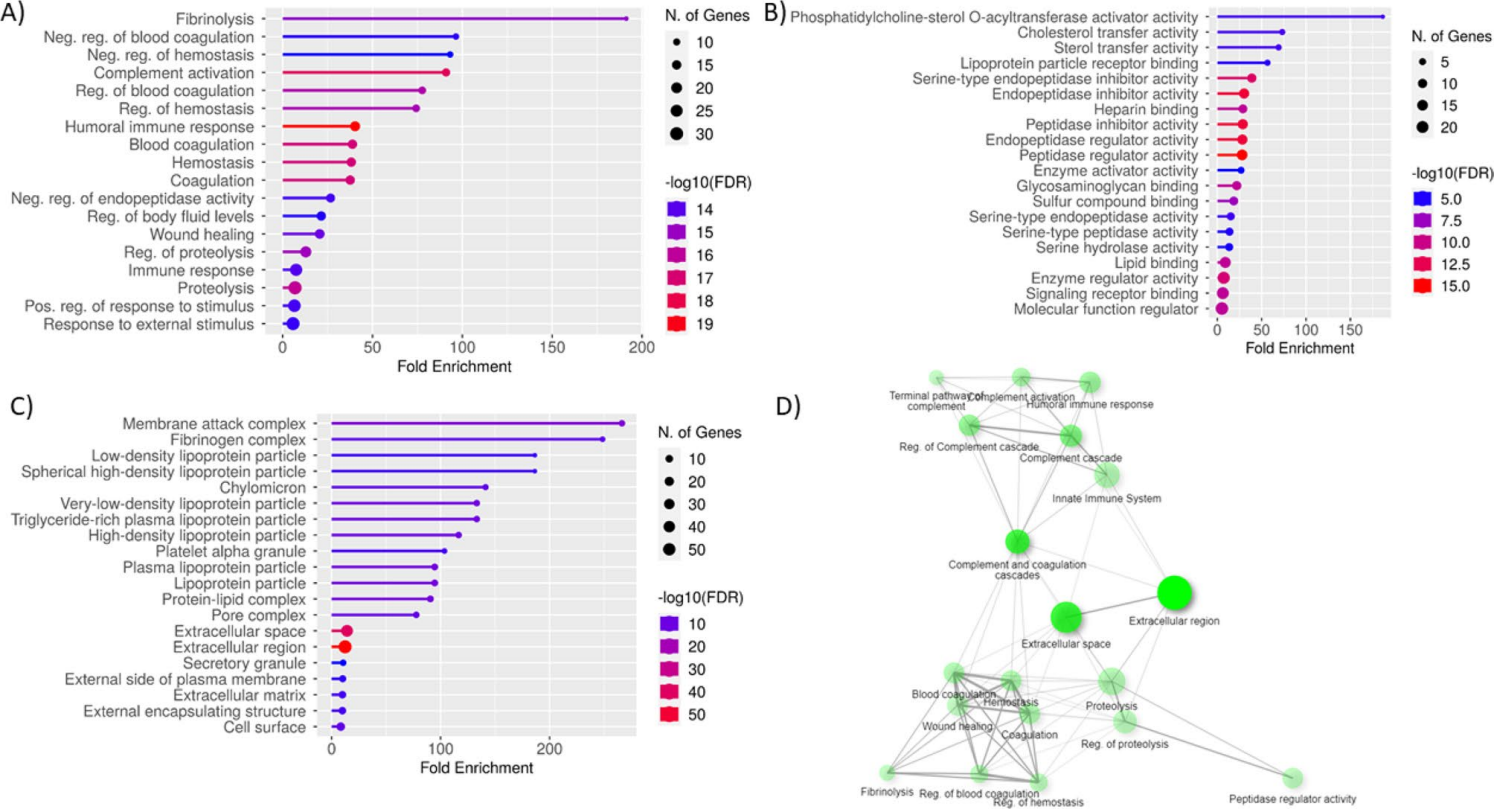
The lollipop charts with top 20 gene ontology (GO) terms with the lowest FDR value are shown for every GO aspect for the proteins with differential abundance between control group, uncomplicated and complicated babesiosis: (**A**) Biological Process; (**B**) Molecular function; (**C**) Cellular component. Terms are sorted by fold enrichment, color corresponds to − log10(FDR) calculated based on nominal P value from the hypergeometric test, and size of circle corresponds to the number of genes belonging to the specific term. (**D**) Network view of the most enriched terms. Two terms (nodes) are connected if they share 20% or more genes, darker nodes are more significantly enriched gene sets, bigger nodes represent larger gene sets and thicker edges represent more overlapped genes.

The top 20 gene ontology (GO) terms with the lowest FDR value are shown for every GO aspect for the genes encoding proteins whose abundance was significantly different between the three groups (Fig. 3). Based on fold enrichment (percentage of genes from the list belonging to a pathway divided by the corresponding percentage in the background) as a measure of effect size, the most overrepresented biological process terms were fibrinolysis, negative regulation of blood coagulation, negative regulation of hemostasis, complement activation and regulation of blood coagulation, while terms which included the highest number of genes were immune response, proteolysis, response to external stimulus and positive regulation of external stimulus (Fig. 3A).

The most overrepresented molecular function terms were phosphatidylcholine-sterol O-acyltransferase activator activity and cholesterol transfer activity, while the majority of genes encoding proteins were included in molecular function regulation, signaling receptor binding and enzyme regulator activity (Fig. 3B). GO terms for the cellular component revealed that the majority of genes belonged to the extracellular region and space, while the most overrepresented were membrane attack complex, fibrinogen complex and low-density lipoprotein particle (Fig. 3C). The network view of the most enriched terms is presented as Fig. 3D.

**Figure 4:**
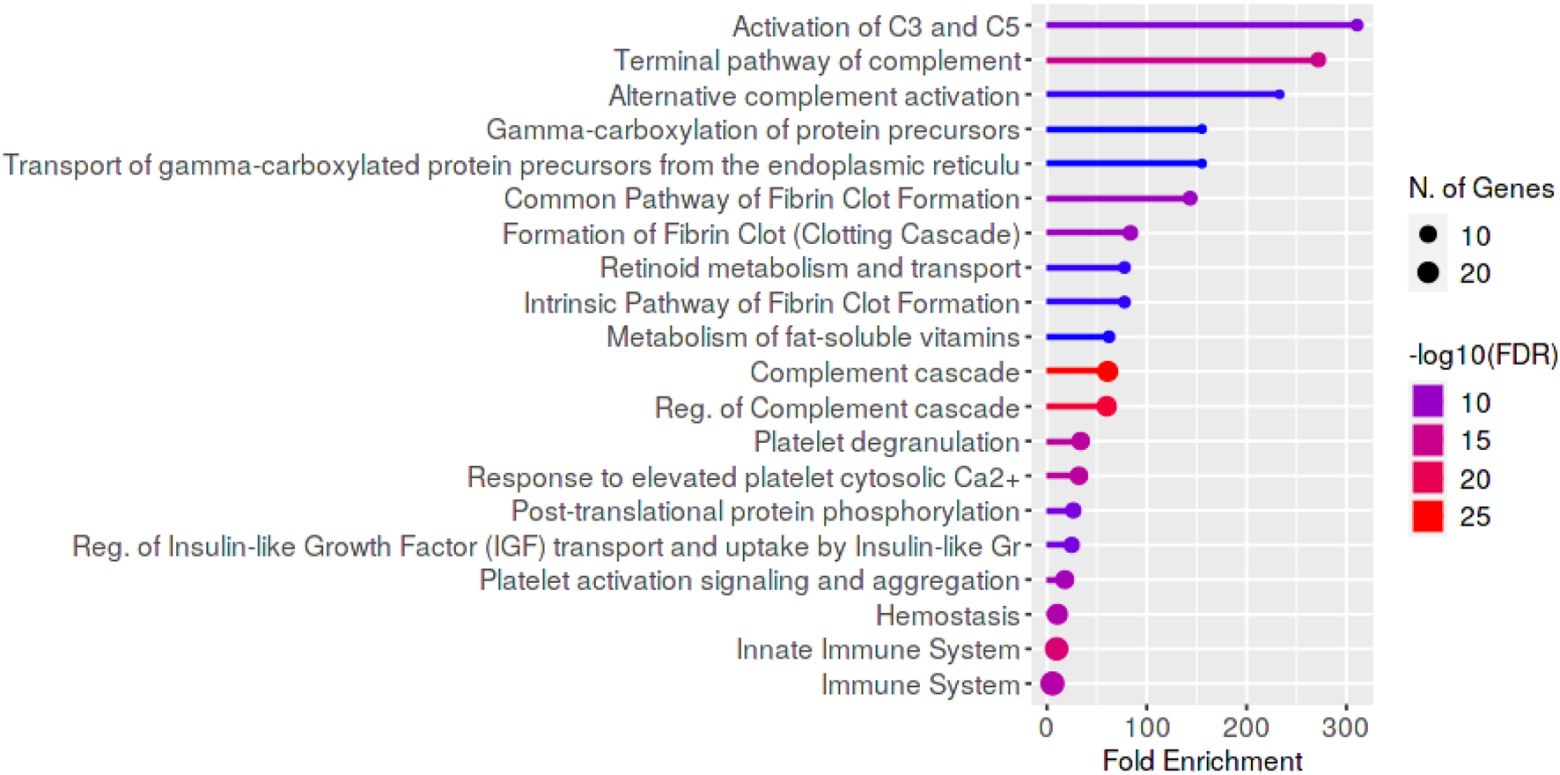
Reactome pathways enriched from proteins with differential abundance between control group, uncomplicated and complicated babesiosis. Pathways are sorted by fold enrichment, color corresponds to − log10(FDR) calculated based on nominal P value from the hypergeometric test, and size of circle corresponds to the number of genes belonging to the specific pathway.

Reactome pathway enrichment analysis revealed that most differentially expressed genes were associated with the immune system, innate immune system, complement cascade and haemostasis, while the most overrepresented pathways were linked to complement, e.g. activation of C3 and C5, terminal pathway of complement and alternative complement activation (Fig. 4).

### Proteomic profiling between uncomplicated and complicated babesiosis

When comparing complicated and uncomplicated babesiosis, 21 proteins showed a difference in abundance between the two groups; 12 had a higher abundance in the complicated group compared to uncomplicated and 9 showed a lower abundance in complicated babesiosis (Table 1).

**Table 1:** Proteins with the significantly differential abundances between dogs with uncomplicated and dogs with complicated babesiosis identified and quantified using tandem mass tags (TMT) proteomics approach.

Accession	Gene ID	Description	*P* value	FDR	log2FC
Proteins with higher abundance in complicated babesiosis compared to uncomplicated babesiosis					
A0A1S7J0A8	CD14	Monocyte differentiation antigen CD14	9.9319E−07	1.26504E−06	0.18
H9GWY3	ITIH4	Inter-alpha-trypsin inhibitor heavy chain 4	8.60294E−09	6.18737E−08	0.51
P18649	APOE	Apolipoprotein E	9.89135E−08	1.60347E−07	0.43
A0A5F4C477	FBLN1	Fibulin-1	4.59563E−05	2.27636E−05	0.39
F6Y713	ORM1	Alpha-1-acid glycoprotein	4.03797E−08	9.00062E−08	0.38
F1PBL4	FGA	Fibrinogen alpha chain	1.72643E−05	1.18406E−05	0.35
A0A5S6CY29	ACTB	Actin, cytoplasmic 1	2.07126E−05	1.23905E−05	0.34
F1PW10	TGFBI	Transforming growth factor-beta-induced protein ig-h3	2.30594E−05	1.26797E−05	0.31
A0A5F4C7X1	CFI	Complement factor I	2.21083E−06	2.07492E−06	0.30
E2RH18	CFP	Complement factor properdin	1.88842E−06	1.98084E−06	0.27
F6XKC0	C8G	Complement C8 gamma chain	1.21139E−05	8.64061E−06	0.20
E2RS79	C2	C3/C5 convertase	5.08565E−06	4.15247E−06	0.19
Proteins with lower abundance in complicated babesiosis compared to uncomplicated babesiosis					
J9NSF9	F2	Activation peptide fragment 1	1.22996E−06	1.37078E−06	− 0.08
J9P430	TF	Beta-1 metal-binding globulin	2.25484E−05	1.26797E−05	− 0.09
A0A5F4BW32	FN1	Fibronectin	2.08E−08	6.18737E−08	− 0.10
F1PG39	ITIH2	Inter-alpha-trypsin inhibitor heavy chain 2	2.02952E−05	1.23905E−05	− 0.17
F1P841	GC	Gc-globulin	9.18069E−06	7.11782E−06	− 0.20
F1PYX9	SERPING1	Serpin family G member 1	2.05778E−06	2.03857E−06	− 0.20
E2QZQ1	TF	Transferrin	6.69568E−05	3.14203E−05	− 0.25
A0A5F4D8W3	PROS1	Vitamin K-dependent protein S	0.000251835	0.000112268	− 0.26
A0A5F4CED7	RBP4	Plasma retinol-binding protein	3.08639E−08	7.86234E−08	− 0.28

**Figure 5:**
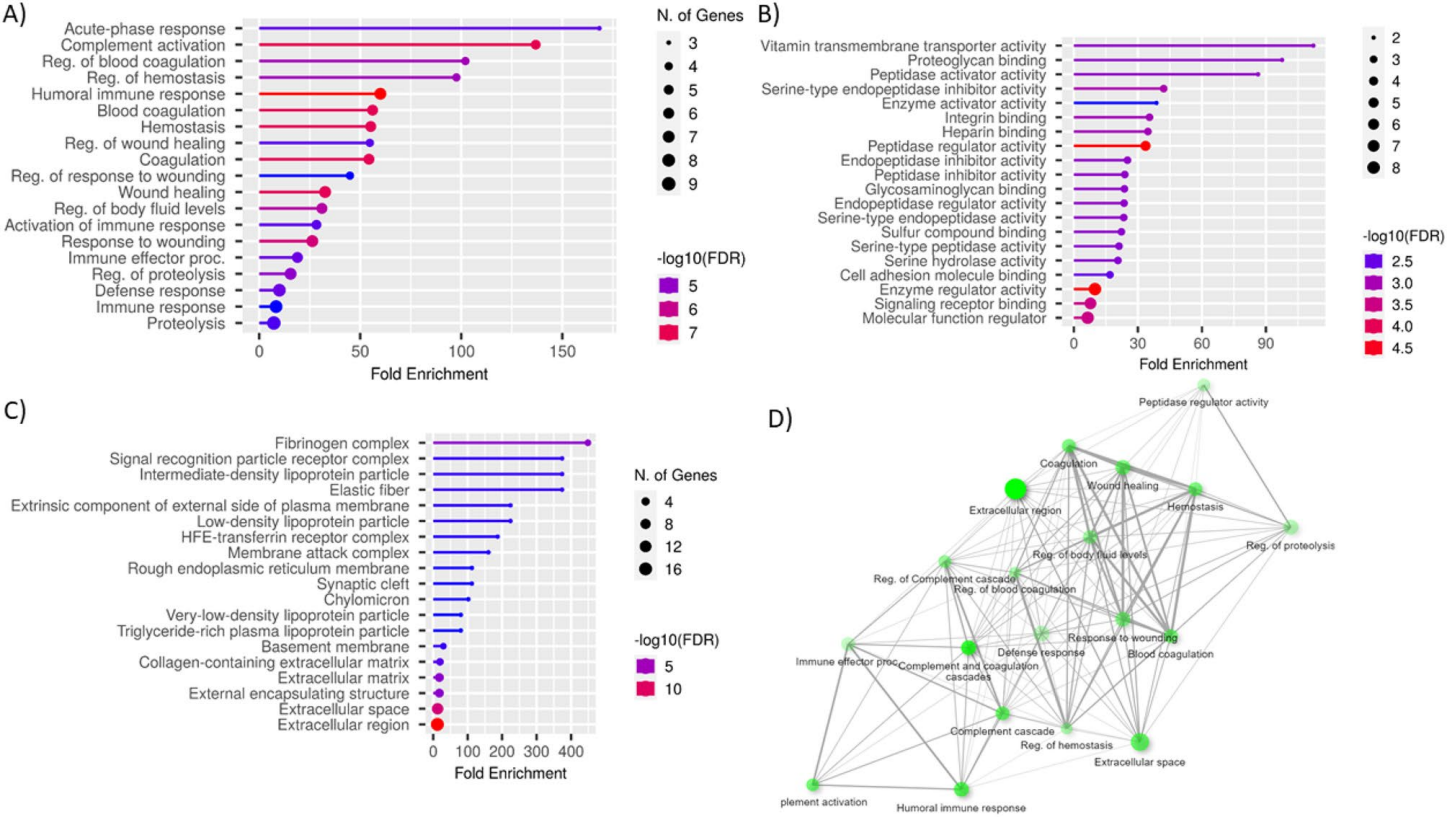
The lollipop charts with top 20 gene ontology (GO) terms with the lowest FDR value are shown for every GO aspect for the proteins with differential abundance between uncomplicated and complicated babesiosis: (**A**) Biological Process; (**B**) Molecular function; (**C**) Cellular component. Terms are sorted by fold enrichment, color corresponds to − log10(FDR) calculated based on nominal P value from the hypergeometric test, and size of circle corresponds to the number of genes belonging to the specific term. (**D**) Network view of the most enriched terms. Two terms (nodes) are connected if they share 20% or more genes, darker nodes are more significantly enriched gene sets, bigger nodes represent larger gene sets and thicker edges represent more overlapped genes.

**Figure 6:**
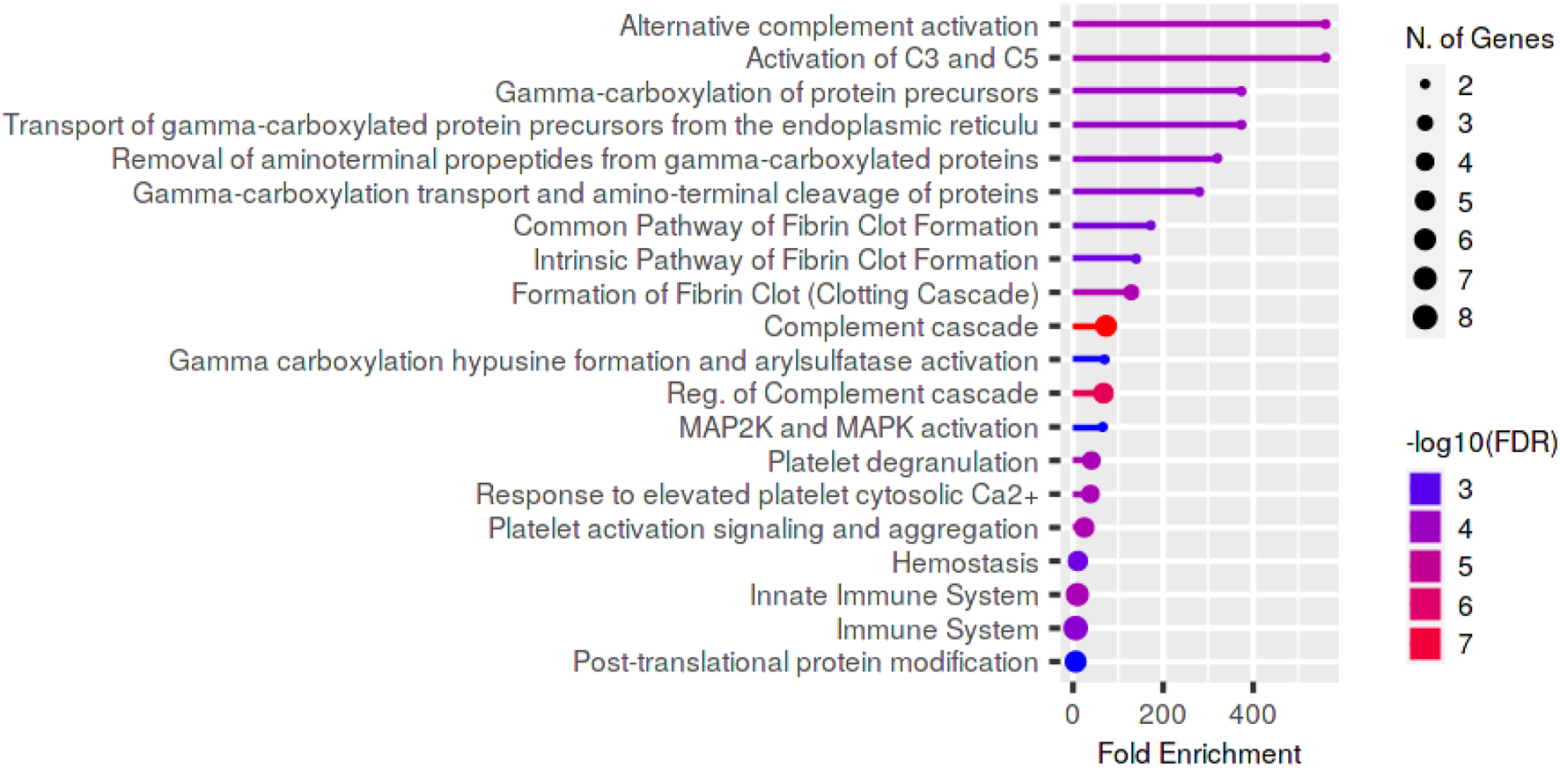
Reactome pathways enriched from proteins with differential abundance between uncomplicated and complicated babesiosis. Pathways are sorted by fold enrichment, color corresponds to − log10(FDR) calculated based on nominal P value from the hypergeometric test, and size of circle corresponds to the number of genes belonging to the specific pathway.

GO enrichment comparing complicated and uncomplicated cases revealed a distinctive set of terms in comparison with the analysis of all proteins, with the most overrepresented biological process terms being acute phase response and complement activation, and for molecular function, overrepresented biological processes were vitamin transmembrane transporter activity and proteoglycan binding (Fig. 5). Reactome pathway enrichment analysis showed a similar profile as shown in the analyses for all the groups investigated, with pathways related to complement being the most overrepresented and the immune system pathway having the highest number of genes represented (Fig. 6).

### Validation

**Figure 7:**
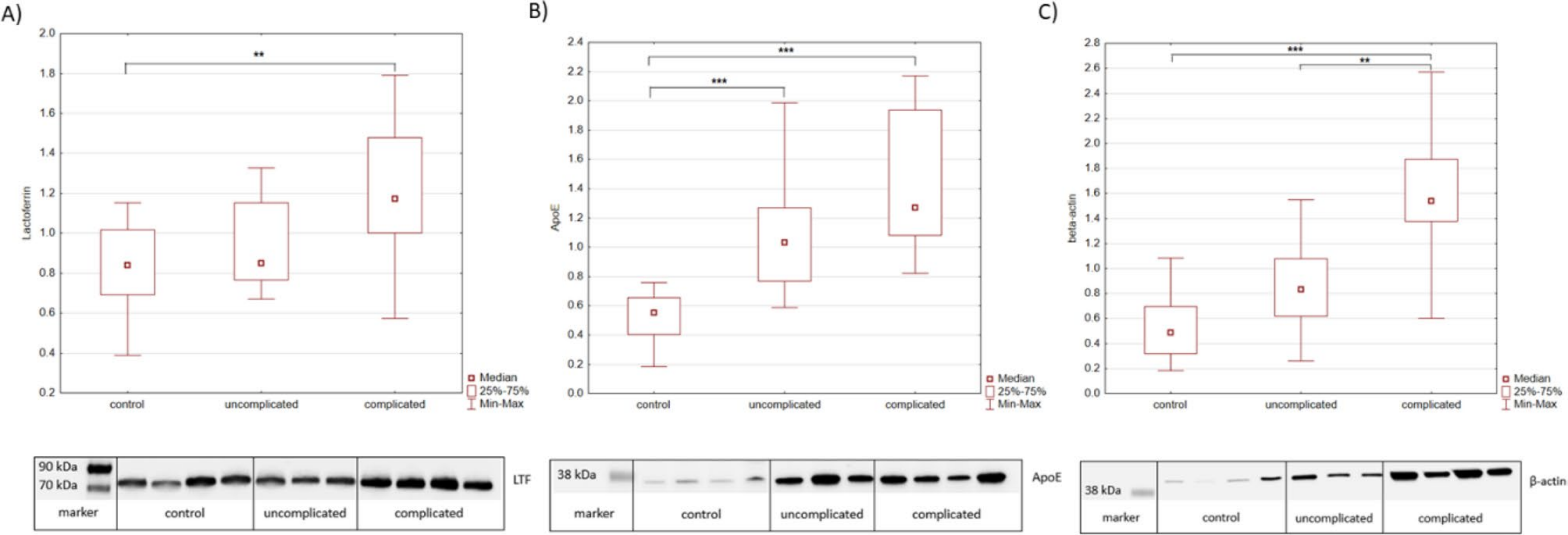
Validation of the proteomics dana. Representative western blot images and relative abundance comparison of (**A**) lactotransferrin (LTF), (**B**) apolipoprotein E (apoE), and (**C**) beta actin between control group, uncomplicated and complicated babesiosis are shown. The abundance of the protein of interest was normalized to the total amount of protein in each lane determined with Ponceau S and quantified using ImageJ software. ^**^ *P* < 0.01, ^***^ *P* < 0.001.

Three selected proteins, namely apolipoprotein E (apoE), beta actin and lactotransferrin (LTF), were validated by western blotting using total protein load after Ponceau S staining as a normalization reference. Results of the Kruskal–Wallis test showed significant differences in relative abundances of all three proteins, although *post-hoc* testing showed slight differences compared to the proteomics data. Relative abundance for LTF was significantly different between the control group and complicated babesiosis (*P* = 0.003), apoE was significantly different between control and uncomplicated babesiosis (*P* < 0.001), as well as between control and complicated babesiosis (*P* < 0.001), while beta actin showed significant difference between control and complicated babesiosis (*P* < 0.001) and between uncomplicated and complicated babesiosis (*P* = 0.004) (Fig. 7). Full (uncropped) western blot images are shown in Supplementary Fig. 1.

## Discussion

A high-throughput label-based proteomic approach was employed for evaluating changes in the proteome in dogs infected with *B. rossi*. To the authors’ knowledge, this is the first time the serum proteome has been evaluated in this complex and multisystemic disease. Compared with the disease caused by other canine *Babesia* parasites, *B. rossi* is clinically the most severe. Previous proteomics studies have used a gel-based proteomic approach^20,21^, while a gel-free TMT-based approach enable more comprehensive identification of proteins and pathways associated with disease pathogenesis and severity. This was a retrospective clinical study and therefore it was not possible to fully adjust for factors such as age, sex, breed and clinical manifestations.

### Proteomic profiling between control group, uncomplicated and complicated babesiosis

There were 78 proteins with significantly differential abundances between the three groups (control, uncomplicated and complicated babesiosis). The top 20 most significantly enriched pathways revealed key differences associated with complement, the innate immune system, haemostasis and lipid metabolism. These pathways reflect and highlight the known pathogenic mechanisms of canine babesiosis such as haemolysis, host response to the inflammation and coagulation^22–24^.

Haemostasis and immune system responses are tightly interrelated pathophysiologic processes with extensive crosstalk^25^. The presence of haemostatic alterations are well documented in canine babesiosis, and further confirmed in this study by significant affected pathways: haemostasis, gamma-carboxylation of protein precursors, transport of gamma-carboxylated protein precursors from the endoplasmic reticulum, common and intrinsic pathway of fibrin clot formation and clotting cascade pathway. Identified proteins involved in these pathways were coagulation factors (F2, F5, F10, F12), serine protease inhibitors (SERPINC1 (antithrombin III), SERPINA5, SERPING1), components of fibrinolysis (FCN1, FGA, FGG) and others. One of the most common and consistent haematological hallmarks of canine babesiosis is thrombocytopenia. Mechanisms for this are still unclear but evidence that platelets play an important role in disease mechanism is growing^23,26,27^. Our work further strengthens this by highlighting platelet-associated pathways, namely platelet degranulation, response to elevated platelet cytosolic Ca^2+^, and platelet activation, signalling and aggregation enriched pathways.

*Babesia rossi* induces a profoundly inflammatory disease with much of the induced pathology associated with a host immune-mediated systemic inflammation^5,28^. Immune system was the single set of pathway that showed the biggest number of differently abundant proteins between all three groups of dogs, including components of complement cascade, acute phase proteins, oxidative stress markers, and components of extracellular matrix. Here we further implicate the central role of host inflammation by showing enriched pathways associated with the immune system, namely the innate immune system, complement cascade, regulation of complement cascade, activation of C3 and C5, terminal pathway of complement and alternative complement activation. Immunohistochemical studies of the pathology of various organs in dogs naturally infected with *B. rossi* have demonstrated a macrophage (a crucial player in the innate immune response) dominated inflammation^29^.

The most overrepresented pathways were related to complement, a major player in the innate immune system. This cascade of soluble proteins, receptors and regulators is activated immediately after encountering the pathogen^30^. Complement is also a well described mediator of haemolysis^31^. Changes in the abundance were found for components of both the classical and alternative pathway (C2, C3, C5, C6, C7, C8A, C8G, C9, CLU, CFP, CPB2, CPN2, SERPING1, CFP, VTN) which lead to the opsonisation and triggering of an inflammatory response. The identification of complement related proteins in the serum of dogs with babesiosis supports the role of this protein complex in the innate immune response to the pathogen.

Acute phase proteins, such as lipopolysaccharide-binding protein (LBP), leucine rich alpha-2-glycoprotein (LRG1), LTF, paraoxonase (PON3), clusterin (CLU), histidine rich glycoprotein (HRG), ceruloplasmin (CP), alpha-1-acid glycoprotein (ORM1), inter-alpha-trypsin inhibitor heavy chain 2 and 4 (ITIH2, ITIH4), were also identified in this study. Most of them have already been previously reported in canine babesiosis^32^. This is however the first time that lactotransferrin has been reported in this disease. Lactotransferrin is an iron-binding glycoprotein of the transferrin family and a major component of the secondary granules of neutrophils. It functions as an iron scavenger and modulator of signaling pathways, leading to the moderation of pro-inflammatory pathway activation during sepsis, thus playing a role in reducing tissue damage^33,34^. Cell-free haemoglobin (a major source of iron) has been shown to play an important role in the pathogenesis and outcome in a canine model of sepsis^35^. Scavenging the free iron that results from haemolysis would thus be an important host protective response^36^. Due to its ability to sequester iron, LTF exhibits a direct antimicrobial activity by destabilizing pathogen membranes and limiting their proliferation and adhesion^37,38^. Its antimicrobial activity was previously evaluated and it was shown to have an inhibitory effect on the in vitro growth of *B. caballi* and *B. equi* (presently reclassified as *Theileria equi*)^39^. The interaction between LTF, red cells and macrophages has been shown to inhibit various intracellular pathogens such as *Toxoplasma, Plasmodium, Leishmania, Trypanosoma*, and *Mycobacterium*^40^. In our study, LTF was significantly more abundant in uncomplicated babesiosis compared to the control group as assessed by proteomic approach, while higher abundance was found in complicated babesiosis compared to control group when assessed by western blotting. One of proposed mechanisms of modulation of the host immune response is by attenuation of the LPS-stimulated TLR-4 signaling pathway by binding to LPS and CD-14^41^. It also activates TLR-4 signaling pathway through NF-kappa-B activation and subsequently induces pro-inflammatory cytokine production^42^. Our data also demonstrated an increased abundances of LBP and CD-14 in babesiosis, underlining the importance of the host inflammatory response and a possible modulating effect of LTF. Lactotransferrin is also a useful biomarker for clinical use due to its resistance to proteolysis and multiple freeze–thaw cycles^43^. The many beneficial roles this protein plays at a molecular and cellular level during infection provides motive to evaluate the role it may play during *B. rossi* infection more closely.

Other significantly affected pathways were related to lipid metabolism, and included proteins such as apolipoproteins (apoA-I, apoA-IV, apoB, apoE, apoM, apoH) and plasma retinol-binding protein (RBP4). A link between babesiosis and lipid metabolism in the host has already been documented by transcriptomics, proteomics, and metabolomics studies^20,44,45^. The importance of lipid metabolism in babesiosis is suggested to be related to the catabolic reaction during the acute phase response in the host. Haemoparasites are unable to synthesize their own lipids and thus rely on uptake from the host^46^. A recent study showed that dogs infected with *B. canis* developed dyslipidemia with altered lipoprotein concentrations and profile, supporting a decreased reverse cholesterol transport from tissues to the liver^47^. The main protein component of high density lipoproteins (HDL), apolipoprotein A-I (apoA-I) participates in the reverse cholesterol transport by promoting cholesterol efflux from tissues. We demonstrated a decreased abundance of apoA-I in complicated and uncomplicated babesiosis compared to control group. This is contrary to previously reported levels in babesiosis caused by *B. canis*, however these studies used gel-based proteomics and radioimmunoassay^20,47^ and hence differences might be present for methodological reasons. In other studies, including canine leishmaniosis^48^ and dogs with sepsis^49^, significantly decreased apoA-I were found. Another lipoprotein of interest was apoE which associates with chylomicrons, chylomicron remnants, very low density lipoproteins (VLDL), intermediate density lipoproteins (IDL), and HDL^50^. This molecule plays a key role in reverse cholesterol transport^51^. Higher abundances of apoE were found in complicated compared to uncomplicated babesiosis, as well as in uncomplicated compared to control group, and in complicated babesiosis compared to controls, as confirmed by western blotting. Lipoproteins are involved in innate and adaptive immune response, the regulation of oxidative pathways and as intermediates in the production of inflammatory mediators^52–54^. Our findings supported interplay between inflammation and lipid metabolism in canine babesiosis, and justify further investigation into different lipoproteins as potential biomarkers and therapeutic targets^55^.

One of the most enriched pathways that emerged in our work was regulation of insulin-like growth factor (IGF) transport and uptake by IGF. This pathway has numerous roles and its expression in the vasculature is affected by interactions with growth factors, cytokines, lipoproteins, reactive oxygen species and haemodynamic forces^56^. A role of this pathway and the proteins involved in it, such as angiotensin (AGT), apoB, thrombin, ITIH2, antithrombin III, fibronectin (FN1), and others, suggested a role for the vascular bed in the pathogenesis of canine babesiosis.

### Proteomic profiling between uncomplicated and complicated babesiosis

The comparison of complicated with uncomplicated babesiosis revealed 21 proteins with difference in abundance between the groups. Twelve had higher abundance in the complicated babesiosis group compared to uncomplicated and nine had lower abundance in complicated babesiosis. The most overrepresented GO terms were acute phase response and complement activation, in contrast with fibrinolysis and regulation of coagulation, which were the most overrepresented when all proteins with differential abundance between three groups were included. This clearly highlights the role of the immune response component with an increase in disease severity. All significantly enriched pathways were related to immune response and haemostasis.

The pathways MAPK2 and MAPK (mitogen-activated protein kinases) activation, which emerged in this comparison, are signal transduction pathways involved in cytokine production, endocytosis, reorganization of the cytoskeleton, gene expression, mitosis, metabolism, motility, survival, apoptosis, and differentiation^57,58^. These serine/threonine kinases convert extracellular stimuli into diverse intracellular responses. Components of the extracellular matrix, such as fibronectin, fibulin-1, vitronectin and actin identified in this study, are also part of this pathway.

Fibulin-1, a calcium-binding glycoprotein, is a member of recently recognized family of extracellular matrix proteins^59^. It has been shown that fibulin-1 has a role in cell adhesion and motility by suppressing the motility promoting activity of fibronectin^60^. It also has a role in haemostasis as it has the ability to bind fibrinogen, promote platelet adhesion and incorporate them into clots^61^. Furthermore, higher fibulin-1 levels were associated with higher soluble urokinase plasminogen activator receptor (suPAR) levels demonstrating its role in vascular dysfunction and extracellular matrix turnover^62^. The vascular endothelium plays a key role in canine babesiosis, with the fibrinolytic system regulating vascular function, local inflammation and tissue remodelling^63,64^. It was also shown that dogs with babesiosis have a higher suPAR concentration compared to healthy dogs^65^. All these findings support cross-talk between the fibrinolytic pathway, processes within the extracellular matrix and inflammation in canine babesiosis. Given its increase in abundance with increase of severity of babesiosis, fibulin-1 could be a potential biomarker for monitoring the disease.

Actin is another protein whose abundance increased with severity of babesiosis. Increase is a result of release into systemic circulation due to vascular and endothelial dysfunction. Increased concentrations were confirmed by both, proteomics and western blotting. A role for this protein has also been proposed during the invasion of the parasite in *B. bovis* infection^66^, as adhesion and motility modulation of *B. bovis* merozoites were driven by fibulin-1 and actin regulation. Vitamin D binding protein (also known as Gc-globulin) binds to actin and has been recognized widely as a protein with markedly decreased concentrations in inflammatory and necrotising diseases, including MODS in canine babesiosis^21,67^. Lower abundance of vitamin D binding protein were associated with the increase of disease severity in the complicated group as confirmed in this study.

Other identified proteins that differentiated uncomplicated from complicated babesiosis were various complement cascade components (CFI, CFP, C2, SERPING1, C8G), extracellular matrix components (TGFBI), acute phase proteins (ORM1, ITIH2, ITIH4, FGA, TF, RBP4) and lipoproteins (apoE). Taken together our findings here demonstrate the association between disease severity and inflammation is central to disease pathogenesis in canine babesiosis. There is also a special accent on derangements in extracellular matrix remodelling.

### Limitations

The most obvious limitation of this study was the fact that it was a retrospective clinical evaluation of a single time point. There was no control of the length of time for which each dog had been sick or for the possibility of clinically silent co-morbidities that may have influenced the outcome. Despite these limitations, the effect of the infection was strong enough to create a clear picture of the proteins and processes dominant in the disease.

Furthermore, to determine whether there are differences in proteomic profiles in response to different Babesia species or different tick-borne parasites, it would be useful to include an additional group of dogs infected with different tick-borne parasites. This approach would provide information on the specificity of the host response, so we propose this as the next step in the investigation.

### Conclusions

The availability of novel omics approaches will accelerate our knowledge of host-parasite interactions and enable progress towards therapeutic tools. Experimental canine sepsis has long been appreciated as a better model than rodent one^68^. The model potential of this canine parasite induced multisystemic inflammatory disease dominated by haemolysis should also be appreciated. It is clear from what we have been able to show that innate immune system activation, haemolysis, haemostasis, lipid metabolism and vessel function are key mechanisms in disease pathogenesis. Elucidation of proteins and pathways involved in canine babesiosis caused by *B. rossi* have established important hallmarks of disease pathogenesis which have the potential to lead to a means of determining disease severity early enough to prevent patient deterioration and could lead to novel therapeutic targets.

## Materials and methods

### Animals

The study was approved by the University of Pretoria Ethics Committee (V0034-14). Informed owner consent was acquired for each case and all procedures were performed in accordance with the approved protocol. The study and all methods were performed in accordance with ARRIVE guidelines^69^. Dogs included were classified into three groups. Healthy dogs were examined at the Clinic as part of a routine examination, and dogs suffering from babesiosis were admitted as patients. The healthy (control) group consisted of 20 dogs with a median age of 21 months (range 12–120 months), 6 females and 14 males. Routine haematologic and biochemical analyses with urinalysis were performed to ensure the health status of the animals, and additionally, none of them had histories of recent illness. The mono-infection with *B. rossi* was confirmed by means of PCR and reverse line blot (PCR RLB) screening for Babesia, Theileria, Hepatozoon and Ehrlichia/Anaplasma species^70^.

Dogs with babesiosis (N = 32) presented clinically ill and had the infection initially diagnosed on a stained peripheral blood smear. The mono-infection with *B. rossi* was confirmed by PCR RLB. The infected dogs were divided into dogs with uncomplicated babesiosis (N = 18) and dogs with complicated babesiosis (N = 14) as previously described^5^. Most of the dogs in this group were mixed breed (N = 5). Other breeds included were Jack Russel Terrier (N = 5), German Shepard (N = 4), and Boerboel (N = 3). The median age was 28 months (range 6–120 months). There were 9 females (3 spayed) and 23 males (4 castrated) altogether, with 5 females (1 spayed) and 13 males (4 castrated) in the uncomplicated group and 4 females (2 spayed) and 10 males in complicated disease group.

Dogs were classified as having complicated babesiosis on the basis of clinical signs and clinical pathology. Clinical signs included: clinical signs of collapse, signs of cerebral disease (seizures, coma) or death within 24 h of admission. Babesiosis was also classified as complicated if one of the following criteria regarding clinical pathology was met: haematocrit less than 0.15 L/L or above 0.5 L/L; urea above 13 mmol/L (reference range: 2.3–8.9 mmol/L); creatinine above 120 µmol/L (reference range: 59–109 µmol/L); total bilirubin above 30 µmol/L (reference range: 1–6.8 µmol/L); blood glucose below 3.3 mmol/L (reference range: 3.3–5.5 mmol/L); lactate above 5 mmol/L (reference range: < 2 mmol/L) or band cell count above 1.5 × 10^9^/L. There were statistically significant differences (*P* < 0.05) between the medians for all these variables between the groups (data not shown, making use of the Mann Whitney U test, SPSS ver. 25). Dogs with uncomplicated disease did not meet any of the above mentioned criteria.

All dogs received standard treatment for canine babesiosis, which included the antibabesial drug diminazene aceturate (3.5 mg/kg intramuscularly). Any complications were treated according to the attending clinician’s discretion. The most commonly provided supportive treatments included packed red cell transfusion, intravenous fluid and electrolyte support and an antiemetic.

### Sample collection and analysis for clinical pathology

Venous blood samples (EDTA and serum) were collected in Vacutainer tubes from the jugular or cephalic veins. Blood gas samples were collected anaerobically into a commercially prepared heparinized syringe (BD A-Line, arterial blood collection syringe, Becton, Dickinson and Company, UK) from the femoral artery. This sample was analysed immediately (Rapidpoint 405, Siemens). Blood glucose was determined on a hand held patient side device (WellionVet GLUCO CALEA Blood Glucose Meter, Germany). The EDTA samples were used for a complete blood count (ADVIA 2120, Siemens, Munich, Germany). Differential white cell counts were performed manually. EDTA anticoagulated blood was also used for DNA extraction for parasite identification by PCR and RLB. Serum was used immediately for serum biochemistry determinations on an automated analyser (Cobas Integra 400 plus, Roche, Basel, Switzerland). Remaining serum samples were archived at − 80 °C in cryovials until used in the proteomic analysis.

### Sample preparation for proteome analysis

Serum proteome analysis was carried out by label-based quantitative approach as described previously, with minor modifications^71^. BCA assay (Thermo Scientific, Rockford, USA) was used for determination of protein concentrations of serum samples. Afterwards, samples (35 µg) and internal standards (a pool of equal protein amount from all samples) were reduced (200 mM dithiothreitol (Sigma-Aldrich, St. Louis, MO, USA), 1 h, 56 °C), alkylated (375 mM iodoacetamide (Sigma-Aldrich, St. Louis, MO, USA), 30 min, in dark) and precipitated with ice-cold acetone (VWR, Radnor, PA, USA) overnight. Protein pellets were subsequently collected by centrifugation (9000 × g, 4 °C), dissolved in 0.1 M triethyl ammonium bicarbonate (Thermo Scientific, Rockford, USA) and digested using 1 μL of trypsin (1 mg/mL, Promega, Madison, USA; trypsin-to-protein ratio 1:35, at 37 °C overnight). TMT sixplex reagents (Thermo Scientific, Rockford, IL, USA) were prepared according to manufacturer’s procedure. An amount of 19 μl of specific TMT label was added to each sample for labelling (60 min, room temperature). The internal standard was labelled with TMT *m/z* 126, while other samples were randomized within TMT sixplex. The reaction was quenched using 5% hydroxylamine (Sigma-Aldrich, St. Louis, MO, USA). Five TMT-modified samples were randomly combined with the internal standard, aliquoted, dried and evaluated by liquid chromatography coupled with tandem mass spectrometry (LC–MS/MS) analysis.

### LC–MS/MS analysis

High resolution LC–MS/MS analysis of TMT-labelled peptides was carried out using an Dionex UltiMate 3000 RSLCnano system (Thermo Fisher Scientific, Gemering, Germany) coupled to a Q Exactive Plus mass spectrometer (Thermo Fisher Scientific, Bremen, Germany). Mobile phase A consisted of 0.1% formic acid in water, and mobile phase B was 0.1% formic acid in 80% ACN. Peptides were dissolved in loading solvent (2% acetonitrile (ACN), 0.1% formic acid) and loaded onto the trap column (C18 PepMap100, 5 μm, 100A, 300 μm × 5 mm), before separation on the analytical column (PepMap™ RSLC C18, 50 cm × 75 μm) using a linear gradient of 5–55% mobile phase B over 120 min, 55–95% for 1 min, held at 95% for 2 min and re-equilibrated at 5% B for 20 min at the flow rate of 300 nL/min. Ionisation was achieved using nanospray Flex ion source (Thermo Fisher Scientific, Bremen, Germany) containing a 10 μm-inner diameter SilicaTip emitter (New Objective, Littleton, MA, USA). The MS operated in positive ion mode using DDA Top8 method. The MS setting was acquired with source voltage of + 2.00 kV, sheath gas of 0 (arbitrary units), auxiliary gas of 0 (arbitrary units), and capillary temperature of 275 °C. S-lens RF level was 60.0 (arbitrary units). Full scan MS spectra were acquired in range from m/z 350.0 to m/z 1800.0 with a resolution of 70,000, 120 ms injection time, AGC target 1 × 10^6^, a ± 2.0 Da isolation window and the dynamic exclusion 30 s. HCD fragmentation was performed at step collision energy (29% and 35% NCE) with a resolution of 17,500 and AGC target of 2 × 10^5^. Precursor ions with unassigned charge state, as well as charge states of + 1 and more than + 7 were excluded from fragmentation.

Acquired MS/MS spectra were analysed using the SEQUEST algorithm implemented into Proteome Discoverer (version 2.3., ThermoFisher Scientific) to obtain protein identification and quantification. Database search against *Canis lupus* FASTA files (downloaded from Uniprot database on October 14th, 2021, 49,889 sequences) was performed according to the following parameters: two trypsin missed cleavage sites, precursor and fragment mass tolerances of 10 ppm and 0.02 Da; carbamidomethyl (C) was set as fixed peptide modification; and oxidation (M), and TMT sixplex (K, peptide N-terminus) as dynamic modifications. The false discovery rate (FDR) for peptide identification was calculated using the Percolator algorithm based on the search results against a decoy database. At least two unique peptides and 1% FDR were required for reporting confidently identified proteins.

### Statistics and bioinformatics

Statistics was performed using R v4.1.2.^72^. Proteins with more than 50% missing values were excluded from the subsequent statistical analysis. As the majority of the identified proteins did not follow a normal distribution (as determined by Shapiro–Wilk test), the Kruskal–Wallis test was used to examine the differences in protein abundance between groups, with *post-hoc* test for pairwise multiple comparisons. Benjamini–Hochberg false discovery rate (FDR) *P* values correction was applied, and proteins were considered statistically significant for FDR < 0.05. Fold change between two groups was calculated as mean (Group2)/mean(Group1) and expressed on log2 scale. Principal component analysis were designed using R package ggplot2 v3.1.1^73^. Hierarchical cluster analysis with dendrograms of the proteins with significantly differential abundances between groups was created using Euclidean as a distance measure and ward as a clustering algorithm, in an open source processing tool MetaboAnalyst^74^.

For bioinformatics analysis, protein accession numbers were first converted into the official gene symbol by UniProtKB ID mapping tool^75^. Enriched gene ontology (GO) classification was performed using ShinyGO tool^76^. REACTOME pathway enrichment analysis using human genome as background was performed and pathways with FDR adjusted *P* value < 0.05 were extracted as significant^77^.

### Validation

Validation of proteomics results was performed by western blotting. For this, the samples (25 μg of total protein) were boiled for 8 min at 95 °C in Laemmli sample buffer and beta-mercaptoethanol and loaded on 12-well Mini-Protean® TGX™ precast polyacrylamide (4–15%) gels (Bio-Rad, Hercules, USA). After electrophoresis (5 min at 50 V, then 70 min at 120 V), proteins were transferred to nitrocellulose membranes on Trans-Blot Turbo Transfer System (Bio-Rad, Hercules, USA) using a pre-programmed three-minute protocol for Mini-Protean® TGX™ gels. Following transfer, the membranes were stained with Ponceau S and membrane images were obtained on Odyssey Fc imager (LI-COR, Bad Homburg, Germany) using a 600 nm channel. Membranes were washed 3 × 5 min with PBS buffer and blocked for 1 h at room temperature with shaking in blocking buffer (Abcam, Cambridge, UK). Subsequently, the membranes were incubated overnight at 4 °C with HRP conjugated primary antibodies for: apolipoprotein E (ApoE) (1:750 in blocking buffer; MyBioSource, San Diego, California, USA, MBS2046336), beta-actin (1:25 000 in blocking buffer; Abcam, Cambridge, UK, AB49900), and lactoferrin (1:750 in blocking buffer; MyBioSource, San Diego, California, USA, MBS2047255). Membranes were then washed 3 × 5 min with TBST buffer and proteins were visualized by chemiluminescence using HRP-chemiluminescence blotting substrate (Radiance Plus, Azure Biosystems, USA) on Odyssey Fc (LI-COR, Bad Homburg, Germany). After visualisation, membranes were washed again 3 × 5 min with TBST and then stripped with WesternSure® ECL Stripping Buffer (LI-COR, Bad Homburg, Germany). The stripped membranes were then incubated with the following primary antibody. The abundance of the protein of interest was normalized to the total amount of protein in each lane determined with Ponceau S. Western blots were quantified using ImageJ software (National Institutes of Health, Bethesda, Maryland, USA). Differences between groups were assessed by Kruskal–Wallis and corresponding *post-hoc* test, with *P* value < 0.05 considered as statistically significant, using GraphPad Prism version 5 software (San Diego, California USA).

## Supplementary Information

Supplementary Information.

## Data Availability

The mass spectrometry proteomics data have been deposited to the ProteomeXchange Consortium via the PRIDE^78^ partner repository with the dataset identifier PXD039022 (https://www.ebi.ac.uk/pride/archive).
